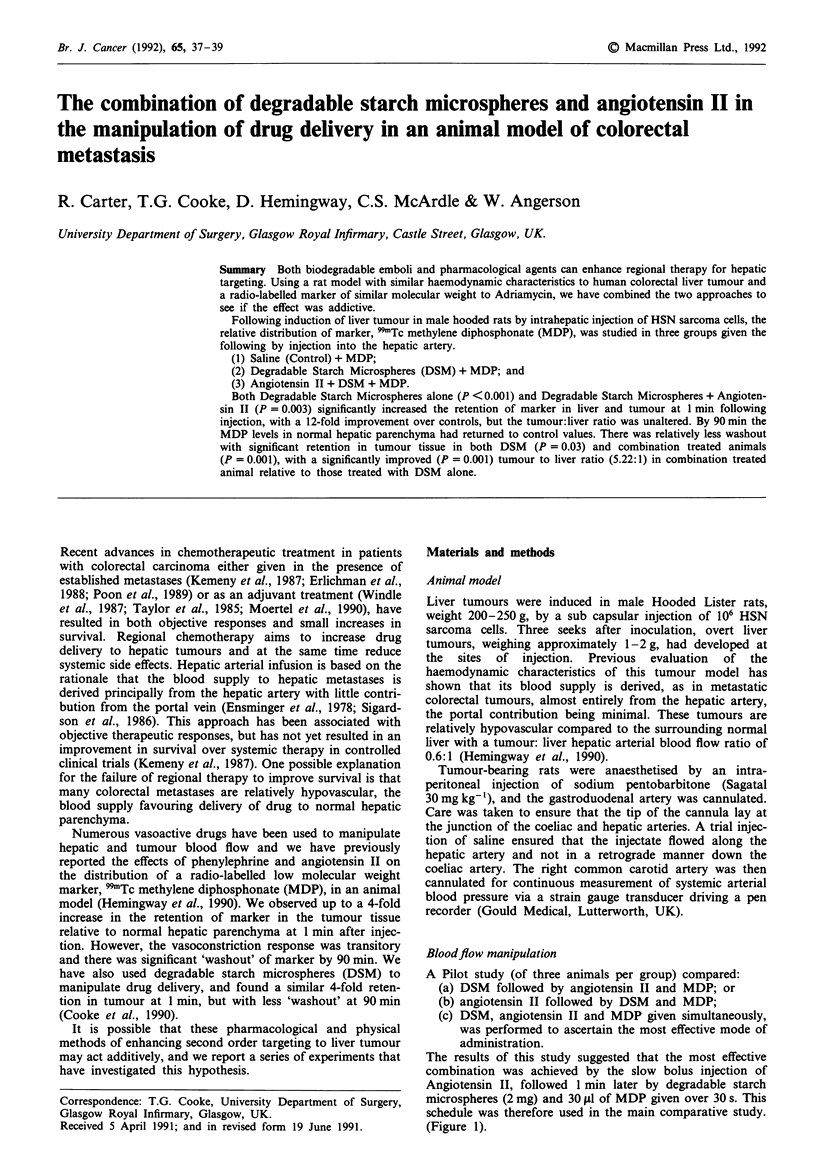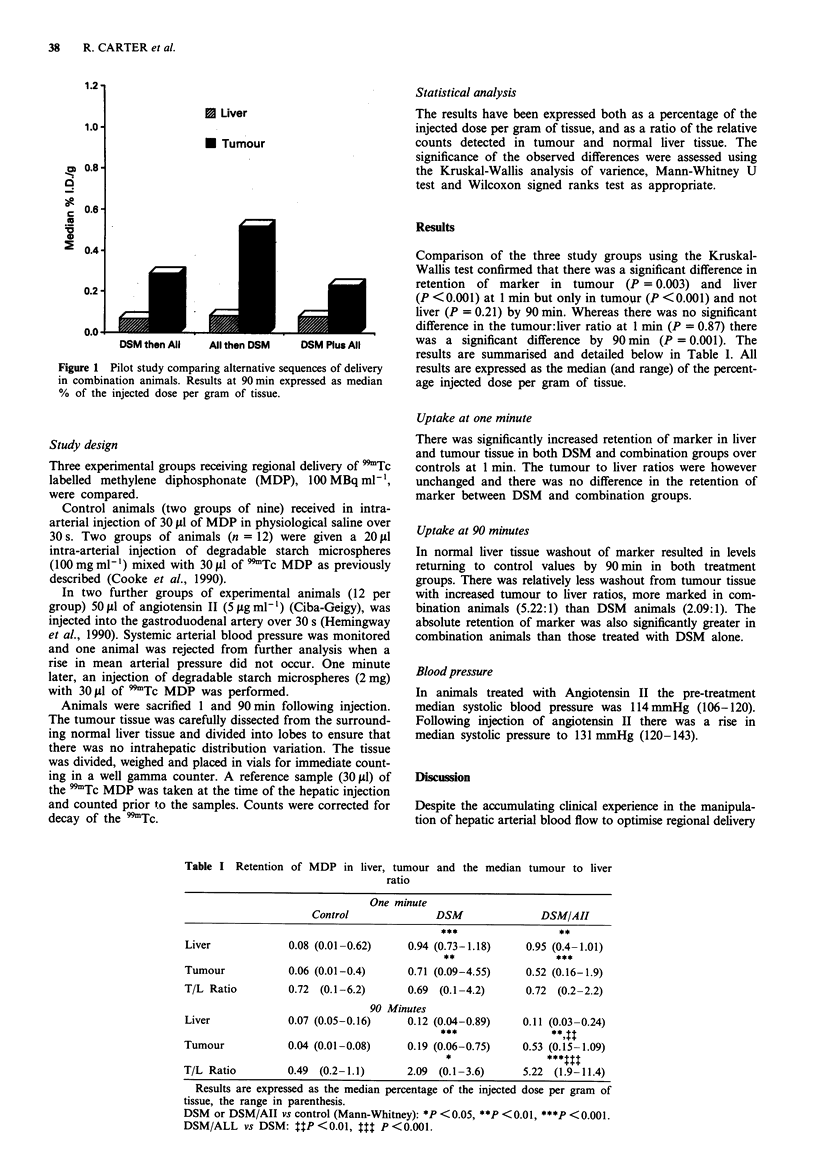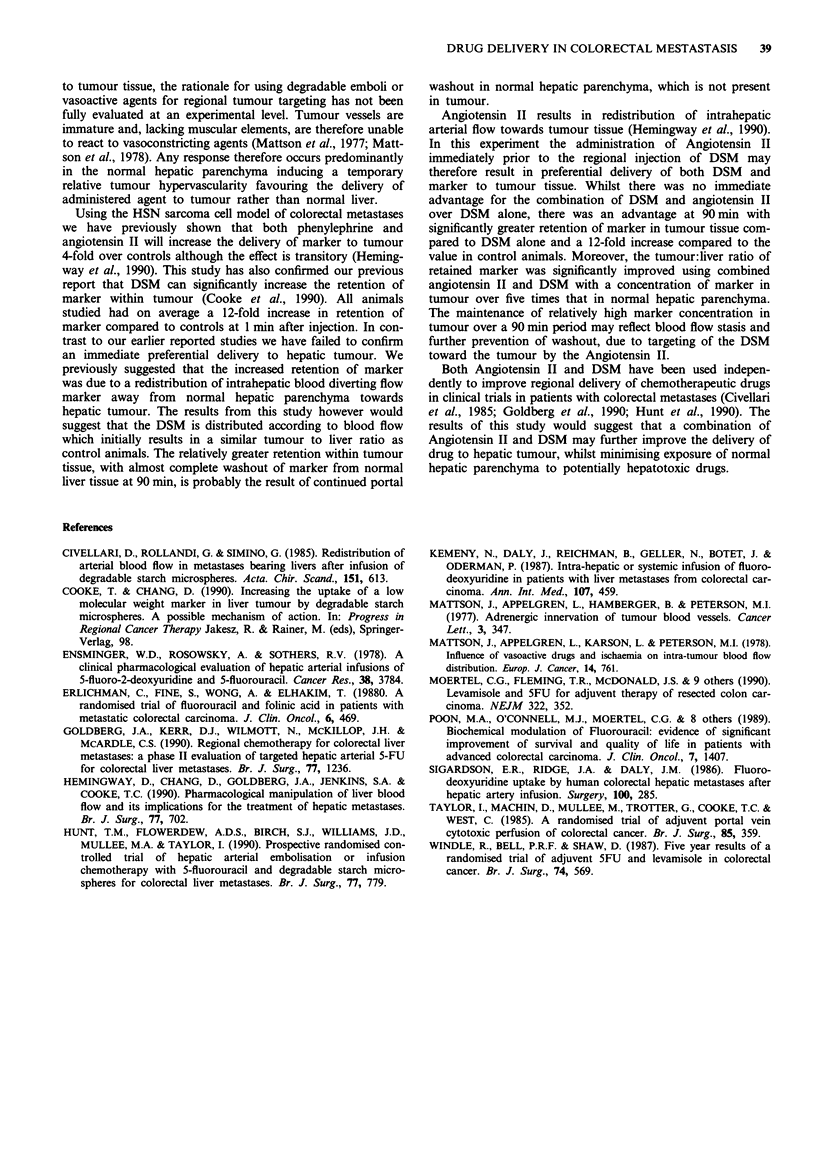# The combination of degradable starch microspheres and angiotensin II in the manipulation of drug delivery in an animal model of colorectal metastasis.

**DOI:** 10.1038/bjc.1992.7

**Published:** 1992-01

**Authors:** R. Carter, T. G. Cooke, D. Hemingway, C. S. McArdle, W. Angerson

**Affiliations:** University Department of Surgery, Glasgow Royal Infirmary, UK.

## Abstract

Both biodegradable emboli and pharmacological agents can enhance regional therapy for hepatic targeting. Using a rat model with similar haemodynamic characteristics to human colorectal liver tumour and a radio-labelled marker of similar molecular weight to Adriamycin, we have combined the two approaches to see if the effect was addictive. Following induction of liver tumour in male hooded rats by intrahepatic injection of HSN sarcoma cells, the relative distribution of marker, 99mTc methylene diphosphonate (MDP), was studied in three groups given the following by injection into the hepatic artery. (1) Saline (Control) + MDP; (2) Degradable Starch Microspheres (DSM) + MDP; and (3) Angiotensin II + DSM + MDP. Both Degradable Starch Microspheres alone (P less than 0.001) and Degradable Starch Microspheres + Angiotensin II (P = 0.003) significantly increased the retention of marker in liver and tumour at 1 min following injection, with a 12-fold improvement over controls, but the tumour:liver ratio was unaltered. By 90 min the MDP levels in normal hepatic parenchyma had returned to control values. There was relatively less washout with significant retention in tumour tissue in both DSM (P = 0.03) and combination treated animals (P = 0.001), with a significantly improved (P = 0.001) tumour to liver ratio (5.22:1) in combination treated animal relative to those treated with DSM alone.


*Br. J. Cancer* (1992), **65**, 37–39 © Macmillan Press Ltd., 1992

### The combination of degradable starch microspheres and angiotensin II in the manipulation of drug delivery in an animal model of colorectal metastasis

R. Carter, T.G. Cooke, D. Hemingway, C.S. McArdle & W. Angerson

*University Department of Surgery, Glasgow Royal Infirmary, Castle Street, Glasgow, UK.*

**Summary** Both biodegradable emboli and pharmacological agents can enhance regional therapy for hepatic targeting. Using a rat model with similar haemodynamic characteristics to human colorectal liver tumour and a radio-labelled marker of similar molecular weight to Adriamycin, we have combined the two approaches to see if the effect was addictive.

Following induction of liver tumour in male hooded rats by intrahepatic injection of HSN sarcoma cells, the relative distribution of marker, $^{99m}$Tc methylene diphosphonate (MDP), was studied in three groups given the following by injection into the hepatic artery.

(1) Saline (Control) + MDP;
(2) Degradable Starch Microspheres (DSM) + MDP; and
(3) Angiotensin II + DSM + MDP.

Both Degradable Starch Microspheres alone ($P < 0.001$) and Degradable Starch Microspheres + Angiotensin II ($P = 0.003$) significantly increased the retention of marker in liver and tumour at 1 min following injection, with a 12-fold improvement over controls, but the tumour:liver ratio was unaltered. By 90 min the MDP levels in normal hepatic parenchyma had returned to control values. There was relatively less washout with significant retention in tumour tissue in both DSM ($P = 0.03$) and combination treated animals ($P = 0.001$), with a significantly improved ($P = 0.001$) tumour to liver ratio (5.22:1) in combination treated animal relative to those treated with DSM alone.

Recent advances in chemotherapeutic treatment in patients with colorectal carcinoma either given in the presence of established metastases (Kemeny *et al.*, 1987; Erlichman *et al.*, 1988; Poon *et al.*, 1989) or as an adjuvant treatment (Windle *et al.*, 1987; Taylor *et al.*, 1985; Moertel *et al.*, 1990), have resulted in both objective responses and small increases in survival. Regional chemotherapy aims to increase drug delivery to hepatic tumours and at the same time reduce systemic side effects. Hepatic arterial infusion is based on the rationale that the blood supply to hepatic metastases is derived principally from the hepatic artery with little contribution from the portal vein (Ensminger *et al.*, 1978; Sigardson *et al.*, 1986). This approach has been associated with objective therapeutic responses, but has not yet resulted in an improvement in survival over systemic therapy in controlled clinical trials (Kemeny *et al.*, 1987). One possible explanation for the failure of regional therapy to improve survival is that many colorectal metastases are relatively hypovascular, the blood supply favouring delivery of drug to normal hepatic parenchyma.

Numerous vasoactive drugs have been used to manipulate hepatic and tumour blood flow and we have previously reported the effects of phenylephrine and angiotensin II on the distribution of a radio-labelled low molecular weight marker, $^{99m}$Tc methylene diphosphonate (MDP), in an animal model (Hemingway *et al.*, 1990). We observed up to a 4-fold increase in the retention of marker in the tumour tissue relative to normal hepatic parenchyma at 1 min after injection. However, the vasoconstriction response was transitory and there was significant 'washout' of marker by 90 min. We have also used degradable starch microspheres (DSM) to manipulate drug delivery, and found a similar 4-fold retention in tumour at 1 min, but with less 'washout' at 90 min (Cooke *et al.*, 1990).

It is possible that these pharmacological and physical methods of enhancing second order targeting to liver tumour may act additively, and we report a series of experiments that have investigated this hypothesis.

Correspondence: T.G. Cooke, University Department of Surgery, Glasgow Royal Infirmary, Glasgow, UK.
Received 5 April 1991; and in revised form 19 June 1991.

### Materials and methods

### *Animal model*

Liver tumours were induced in male Hooded Lister rats, weight 200–250 g, by a sub capsular injection of $10^6$ HSN sarcoma cells. Three seeks after inoculation, overt liver tumours, weighing approximately 1–2 g, had developed at the sites of injection. Previous evaluation of the haemodynamic characteristics of this tumour model has shown that its blood supply is derived, as in metastatic colorectal tumours, almost entirely from the hepatic artery, the portal contribution being minimal. These tumours are relatively hypovascular compared to the surrounding normal liver with a tumour: liver hepatic arterial blood flow ratio of 0.6:1 (Hemingway *et al.*, 1990).

Tumour-bearing rats were anaesthetised by an intraperitoneal injection of sodium pentobarbitone (Sagatal 30 mg kg$^{-1}$), and the gastroduodenal artery was cannulated. Care was taken to ensure that the tip of the cannula lay at the junction of the coeliac and hepatic arteries. A trial injection of saline ensured that the injectate flowed along the hepatic artery and not in a retrograde manner down the coeliac artery. The right common carotid artery was then cannulated for continuous measurement of systemic arterial blood pressure via a strain gauge transducer driving a pen recorder (Gould Medical, Lutterworth, UK).

### *Blood flow manipulation*

A Pilot study (of three animals per group) compared:
(a) DSM followed by angiotensin II and MDP; or
(b) angiotensin II followed by DSM and MDP;
(c) DSM, angiotensin II and MDP given simultaneously, was performed to ascertain the most effective mode of administration.

The results of this study suggested that the most effective combination was achieved by the slow bolus injection of Angiotensin II, followed 1 min later by degradable starch microspheres (2 mg) and 30 µl of MDP given over 30 s. This schedule was therefore used in the main comparative study. (Figure 1).

**Figure 1** Pilot study comparing alternative sequences of delivery in combination animals. Results at 90 min expressed as median % of the injected dose per gram of tissue.

*Study design*

Three experimental groups receiving regional delivery of $^{99m}$Tc labelled methylene diphosphonate (MDP), 100 MBq ml$^{-1}$, were compared.

Control animals (two groups of nine) received in intra-arterial injection of 30 µl of MDP in physiological saline over 30 s. Two groups of animals ($n$ = 12) were given a 20 µl intra-arterial injection of degradable starch microspheres (100 mg ml$^{-1}$) mixed with 30 µl of $^{99m}$Tc MDP as previously described (Cooke *et al.*, 1990).

In two further groups of experimental animals (12 per group) 50 µl of angiotensin II (5 µg ml$^{-1}$) (Ciba-Geigy), was injected into the gastroduodenal artery over 30 s (Hemingway *et al.*, 1990). Systemic arterial blood pressure was monitored and one animal was rejected from further analysis when a rise in mean arterial pressure did not occur. One minute later, an injection of degradable starch microspheres (2 mg) with 30 µl of $^{99m}$Tc MDP was performed.

Animals were sacrified 1 and 90 min following injection. The tumour tissue was carefully dissected from the surrounding normal liver tissue and divided into lobes to ensure that there was no intrahepatic distribution variation. The tissue was divided, weighed and placed in vials for immediate counting in a well gamma counter. A reference sample (30 µl) of the $^{99m}$Tc MDP was taken at the time of the hepatic injection and counted prior to the samples. Counts were corrected for decay of the $^{99m}$Tc.

*Statistical analysis*

The results have been expressed both as a percentage of the injected dose per gram of tissue, and as a ratio of the relative counts detected in tumour and normal liver tissue. The significance of the observed differences were assessed using the Kruskal-Wallis analysis of varience, Mann-Whitney U test and Wilcoxon signed ranks test as appropriate.

### Results

Comparison of the three study groups using the Kruskal-Wallis test confirmed that there was a significant difference in retention of marker in tumour ($P = 0.003$) and liver ($P < 0.001$) at 1 min but only in tumour ($P < 0.001$) and not liver ($P = 0.21$) by 90 min. Whereas there was no significant difference in the tumour:liver ratio at 1 min ($P = 0.87$) there was a significant difference by 90 min ($P = 0.001$). The results are summarised and detailed below in Table I. All results are expressed as the median (and range) of the percentage injected dose per gram of tissue.

*Uptake at one minute*

There was significantly increased retention of marker in liver and tumour tissue in both DSM and combination groups over controls at 1 min. The tumour to liver ratios were however unchanged and there was no difference in the retention of marker between DSM and combination groups.

*Uptake at 90 minutes*

In normal liver tissue washout of marker resulted in levels returning to control values by 90 min in both treatment groups. There was relatively less washout from tumour tissue with increased tumour to liver ratios, more marked in combination animals (5.22:1) than DSM animals (2.09:1). The absolute retention of marker was also significantly greater in combination animals than those treated with DSM alone.

*Blood pressure*

In animals treated with Angiotensin II the pre-treatment median systolic blood pressure was 114 mmHg (106–120). Following injection of angiotensin II there was a rise in median systolic pressure to 131 mmHg (120–143).

### Discussion

Despite the accumulating clinical experience in the manipulation of hepatic arterial blood flow to optimise regional delivery

**Table I** Retention of MDP in liver, tumour and the median tumour to liver ratio

| | *Control* | *DSM* | *DSM/AII* |
|---|---|---|---|
| | | *One minute* | |
| Liver | 0.08 (0.01–0.62) | *** 0.94 (0.73–1.18) | ** 0.95 (0.4–1.01) |
| Tumour | 0.06 (0.01–0.4) | ** 0.71 (0.09–4.55) | *** 0.52 (0.16–1.9) |
| T/L Ratio | 0.72 (0.1–6.2) | 0.69 (0.1–4.2) | 0.72 (0.2–2.2) |
| | | *90 Minutes* | |
| Liver | 0.07 (0.05–0.16) | 0.12 (0.04–0.89) | 0.11 (0.03–0.24) |
| Tumour | 0.04 (0.01–0.08) | *** 0.19 (0.06–0.75) | **,‡‡ 0.53 (0.15–1.09) |
| T/L Ratio | 0.49 (0.2–1.1) | * 2.09 (0.1–3.6) | ***‡‡‡ 5.22 (1.9–11.4) |

Results are expressed as the median percentage of the injected dose per gram of tissue, the range in parenthesis.
DSM or DSM/AII *vs* control (Mann-Whitney): *$P < 0.05$, **$P < 0.01$, ***$P < 0.001$.
DSM/ALL *vs* DSM: ‡‡$P < 0.01$, ‡‡‡ $P < 0.001$.

to tumour tissue, the rationale for using degradable emboli or vasoactive agents for regional tumour targeting has not been fully evaluated at an experimental level. Tumour vessels are immature and, lacking muscular elements, are therefore unable to react to vasoconstricting agents (Mattson *et al.*, 1977; Mattson *et al.*, 1978). Any response therefore occurs predominantly in the normal hepatic parenchyma inducing a temporary relative tumour hypervascularity favouring the delivery of administered agent to tumour rather than normal liver.

Using the HSN sarcoma cell model of colorectal metastases we have previously shown that both phenylephrine and angiotensin II will increase the delivery of marker to tumour 4-fold over controls although the effect is transitory (Hemingway *et al.*, 1990). This study has also confirmed our previous report that DSM can significantly increase the retention of marker within tumour (Cooke *et al.*, 1990). All animals studied had on average a 12-fold increase in retention of marker compared to controls at 1 min after injection. In contrast to our earlier reported studies we have failed to confirm an immediate preferential delivery to hepatic tumour. We previously suggested that the increased retention of marker was due to a redistribution of intrahepatic blood diverting flow marker away from normal hepatic parenchyma towards hepatic tumour. The results from this study however would suggest that the DSM is distributed according to blood flow which initially results in a similar tumour to liver ratio as control animals. The relatively greater retention within tumour tissue, with almost complete washout of marker from normal liver tissue at 90 min, is probably the result of continued portal washout in normal hepatic parenchyma, which is not present in tumour.

Angiotensin II results in redistribution of intrahepatic arterial flow towards tumour tissue (Hemingway *et al.*, 1990). In this experiment the administration of Angiotensin II immediately prior to the regional injection of DSM may therefore result in preferential delivery of both DSM and marker to tumour tissue. Whilst there was no immediate advantage for the combination of DSM and angiotensin II over DSM alone, there was an advantage at 90 min with significantly greater retention of marker in tumour tissue compared to DSM alone and a 12-fold increase compared to the value in control animals. Moreover, the tumour:liver ratio of retained marker was significantly improved using combined angiotensin II and DSM with a concentration of marker in tumour over five times that in normal hepatic parenchyma. The maintenance of relatively high marker concentration in tumour over a 90 min period may reflect blood flow stasis and further prevention of washout, due to targeting of the DSM toward the tumour by the Angiotensin II.

Both Angiotensin II and DSM have been used independently to improve regional delivery of chemotherapeutic drugs in clinical trials in patients with colorectal metastases (Civellari *et al.*, 1985; Goldberg *et al.*, 1990; Hunt *et al.*, 1990). The results of this study would suggest that a combination of Angiotensin II and DSM may further improve the delivery of drug to hepatic tumour, whilst minimising exposure of normal hepatic parenchyma to potentially hepatotoxic drugs.

### References

CIVELLARI, D., ROLLANDI, G. & SIMINO, G. (1985). Redistribution of arterial blood flow in metastases bearing livers after infusion of degradable starch microspheres. *Acta. Chir. Scand.*, **151**, 613.

COOKE, T. & CHANG, D. (1990). Increasing the uptake of a low molecular weight marker in liver tumour by degradable starch microspheres. A possible mechanism of action. In: *Progress in Regional Cancer Therapy* Jakesz, R. & Rainer, M. (eds), Springer-Verlag, 98.

ENSMINGER, W.D., ROSOWSKY, A. & SOTHERS, R.V. (1978). A clinical pharmacological evaluation of hepatic arterial infusions of 5-fluoro-2-deoxyuridine and 5-fluorouracil. *Cancer Res.*, **38**, 3784.

ERLICHMAN, C., FINE, S., WONG, A. & ELHAKIM, T. (19880. A randomised trial of fluorouracil and folinic acid in patients with metastatic colorectal carcinoma. *J. Clin. Oncol.*, **6**, 469.

GOLDBERG, J.A., KERR, D.J., WILMOTT, N., MCKILLOP, J.H. & MCARDLE, C.S. (1990). Regional chemotherapy for colorectal liver metastases: a phase II evaluation of targeted hepatic arterial 5-FU for colorectal liver metastases. *Br. J. Surg.*, **77**, 1236.

HEMINGWAY, D., CHANG, D., GOLDBERG, J.A., JENKINS, S.A. & COOKE, T.C. (1990). Pharmacological manipulation of liver blood flow and its implications for the treatment of hepatic metastases. *Br. J. Surg.*, **77**, 702.

HUNT, T.M., FLOWERDEW, A.D.S., BIRCH, S.J., WILLIAMS, J.D., MULLEE, M.A. & TAYLOR, I. (1990). Prospective randomised controlled trial of hepatic arterial embolisation or infusion chemotherapy with 5-fluorouracil and degradable starch microspheres for colorectal liver metastases. *Br. J. Surg.*, **77**, 779.

KEMENY, N., DALY, J., REICHMAN, B., GELLER, N., BOTET, J. & ODERMAN, P. (1987). Intra-hepatic or systemic infusion of fluorodeoxyuridine in patients with liver metastases from colorectal carcinoma. *Ann. Int. Med.*, **107**, 459.

MATTSON, J., APPELGREN, L., HAMBERGER, B. & PETERSON, M.I. (1977). Adrenergic innervation of tumour blood vessels. *Cancer Lett.*, **3**, 347.

MATTSON, J., APPELGREN, L., KARSON, L. & PETERSON, M.I. (1978). Influence of vasoactive drugs and ischaemia on intra-tumour blood flow distribution. *Europ. J. Cancer*, **14**, 761.

MOERTEL, C.G., FLEMING, T.R., MCDONALD, J.S. & 9 others (1990). Levamisole and 5FU for adjuvent therapy of resected colon carcinoma. *NEJM* 322, 352.

POON, M.A., O'CONNELL, M.J., MOERTEL, C.G. & 8 others (1989). Biochemical modulation of Fluorouracil: evidence of significant improvement of survival and quality of life in patients with advanced colorectal carcinoma. *J. Clin. Oncol.*, **7**, 1407.

SIGARDSON, E.R., RIDGE, J.A. & DALY, J.M. (1986). Fluorodeoxyuridine uptake by human colorectal hepatic metastases after hepatic artery infusion. *Surgery*, **100**, 285.

TAYLOR, I., MACHIN, D., MULLEE, M., TROTTER, G., COOKE, T.C. & WEST, C. (1985). A randomised trial of adjuvent portal vein cytotoxic perfusion of colorectal cancer. *Br. J. Surg.*, **85**, 359.

WINDLE, R., BELL, P.R.F. & SHAW, D. (1987). Five year results of a randomised trial of adjuvent 5FU and levamisole in colorectal cancer. *Br. J. Surg.*, **74**, 569.